# An Alternative One-Stage Exchange Arthroplasty Technique: For the Chronic Infected Total Hip

**DOI:** 10.7759/cureus.11138

**Published:** 2020-10-24

**Authors:** Keith Kotecki, Victor Hoang, Daniel LeCavalier, Michael Bradford

**Affiliations:** 1 Orthopaedic Surgery, Valley Hospital Medical Center, Las Vegas, USA; 2 Orthopaedic Surgery, Nevada Orthopedic and Spine Center, Las Vegas, USA

**Keywords:** tha, total hip arthroplasty, pji, prosthetic joint infection, 1-stage exchange, chronic hip infection

## Abstract

Background

There are various algorithms for the treatment of prosthetic joint infections (PJI). Currently, a two-stage hip exchange is considered the “gold standard” of care for treatment of chronic hip PJIs. However, there has been recent debate whether a one- or two-stage exchange offers the correct treatment. One-stage exchange arthroplasty has particularly gained interest due to less morbidity, mortality, and functional impairment.

Methods

In a retrospective case series, the outcome of patients with chronic hip PJIs treated with our one-stage exchange arthroplasty was analyzed. Between January 2015 and January 2020, eight patients underwent a one-stage exchange hip arthroplasty by a single surgeon at a single institution for a chronically infected total hip arthroplasty (THA). Original diagnosis of PJI was made in accordance with the 2011 version of the Musculoskeletal Infection Society (MSIS) criteria. The femoral stem was cemented with antibiotic-impregnated cement, and the polyethylene acetabular liner was cemented directly onto the acetabular bone with antibiotic-impregnated cement.

Results

Of the eight patients, three were female and five were male with a mean age of 70.5 years (SD 11.2, range 53-87). Six patients (75%) had infection eradication with retention of a stable implant and no additional surgery at a mean follow-up of 35.7 months (range 17-50). One patient (12.5%) underwent closed reduction for a dislocated THA at one month; however, this patient remained infection-free at the most recent follow-up of 41 months. One patient (12.5%) who was the oldest patient (87 years) died 18 days postoperatively. Overall, all living patients (87.5%) retained their one-stage exchange THA. One patient (12.5%, CI 95% 0.3-52.7) required additional surgery in the form of a closed reduction and zero patients (0.0%, CI 95% 0.0-36.9) required additional open surgery.

Conclusion

Single-stage exchange arthroplasty with an antibiotic-impregnated cemented femoral stem and antibiotic-impregnated cemented polyethylene acetabular liner may be a useful option for the treatment of chronic hip PJIs. Our case series provides evidence that infection eradication and function preservation are possible using our one-stage exchange arthroplasty technique in a chronically infected THA. However, a multi-center study with randomization is necessary to further validate our results.

## Introduction

Prosthetic joint infections (PJI) are a devastating complication after total hip arthroplasty (THA) procedures that remains the leading cause of failure within five years [1]. Despite advancements in PJI prevention, THA infection rates are reported between 1 and 3% [2,3]. By the year 2030, primary THA is projected to increase by 171% to 635,000 surgeries annually and revision THA is projected to increase by 142% to 72,000 surgeries annually [4]. This creates the potential for as many as 19,050 infected primary hips and 2,160 infected revision hips by 2030.

A chronic PJI is defined as infection greater than four weeks postoperatively or greater than three weeks after the development of symptoms from haematogenous spread [5]. This definition derived from the theory that bacteria produce biofilms on implants after three weeks requiring their removal [6-8]. As such, this necessitates removal of all infected organic and non-organic material followed by immediate or delayed re-implantation [9].

Currently, a two-stage exchange hip revision is considered the “gold standard” of care for treatment of chronic PJIs [10]. However, reported failure rates after a two-stage hip revision range from 5 to 18% [11,12]. Thus, there has been debate whether a one-stage exchange or two-stage exchange offers the correct treatment [13]. One-stage exchange arthroplasty has particularly gained interest due to less morbidity, mortality, and functional impairment [14].

This study describes an alternative one-stage exchange arthroplasty technique for the treatment of chronic prosthetic hip infections. To the best of our knowledge a one-stage exchange where the polyethylene liner is directly cemented into the acetabulum without a metal cup has yet to be described in the literature. Thus, the primary purpose of the study is to describe our technique and secondary report initial results in a case series. 

## Materials and methods

The study was approved by the Touro University of Nevada Institutional Review Board (IRB) located in Las Vegas, Nevada prior to the initiation of the study. We conducted a retrospective search of our centers database of 83 patients who underwent a revision PJI by a single surgeon between January 2015 and January 2020. Of the initial 83 patients, 51 patients underwent a revision total knee arthroplasty for an acute or chronic infected total knee, 24 patients underwent a DAIR (Debridement, Antibiotics, and Implant Retention) procedure for an acutely infected total hip, and eight patients underwent a one-stage exchange arthroplasty for a chronically infected total hip. All patients diagnosed with a chronic hip PJI treated by the lead surgeon (MB) at a single institution between January 2015 and January 2020 were included. A chronic hip PJI was defined as the development of a joint infection greater than six weeks postoperatively. Both infected chronic primary and revision hips were included in this study. The lead surgeon principally treats chronic infected hips with the described one-stage exchange technique; thus, a two-stage exchange cohort was unable to be included for comparison. Demographic characteristics and cohort description are presented in Table 1.

**Table 1:** Demographics, microorganisms, perioperative variables, and clinical outcomes Time to latest infection-free follow is reported; DAIR = debridement, antibiotics, and implant retention; THA = total hip arthroplasty; ORIF = open reduction internal fixation; Hemi = hemiarthroplasty; BMI = body mass index; N/A = not applicable; MSSA = methicillin-sensitive Staphylococcus aures; MRSA = methicillin-resistant Staphylococcus aures; GBS = group B Streptococcus

Patient Number	Age (years)	Sex	Smoker (Prior to surgery, Current smoker at time of surgery)	Diabetic	BMI	Side	Prior surgery performed	Interval between prior surgery and one-stage (months)	Infecting organism	Outcome	Time to further surgery (months)	Infection free follow up (months)
1	53	Female	No	Yes	43.36	Left	DAIR	4	MSSA	Retention	N/A	50
2	80	Male	No	Yes	32.71	Right	Revision THA with ORIF	8	Coagulase-negative staph.	Retention	N/A	48
3	80	Male	No	No	25.2	Right	Primary THA	144	MRSA	Retention, but closed reduction for dislocation	1	41
4	65	Female	Current	No	17.51	Right	Primary THA	60	Strep. acalactiae (GBS)	Retention	N/A	37
5	57	Female	Current	No	16.72	Left	DAIR	3	MRSA	Retention	N/A	32
6	87	Male	No	No	21.07	Right	Bipolar Hemi	2	MRSA	Deceased	N/A	Deceased 18 days postop
7	67	Male	Prior	Yes	28.15	Left	Primary THA	132	MSSA	Retention	N/A	30
8	75	Male	No	No	24.26	Left	Revision THA	15	Cornyebacterium striatum	Retention	N/A	17

**Figure 1:**
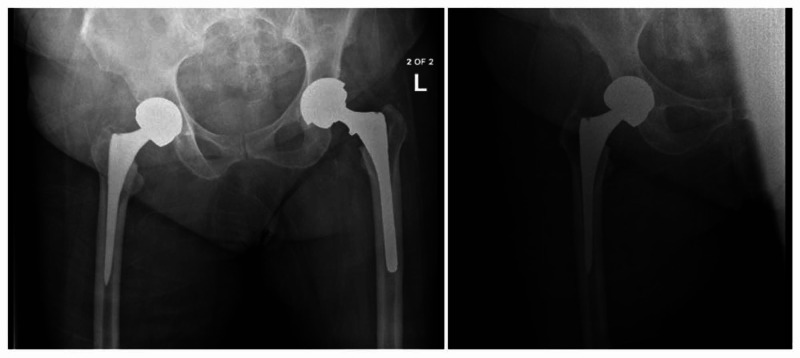
Preoperative plain radiograph to assess symptomatic right primary total hip arthroplasty (THA) that was performed by another surgeon.

Patients that presented with infectious symptoms after a total hip arthroplasty were evaluated for possible PJI. Symptoms such as pain, local erythema, effusion, warmth, and fever were considered suspicious for PJI. Plain radiographs were obtained and compared to previous radiographs to assess implant position, polyethylene wear, and osteolysis (Figure 1). A technetium-99m bone scan was used to evaluate for loosening or infection. Preoperative laboratory studies included a complete blood count (CBC), complete metabolic panel (CMP), erythrocyte sedimentation rate (ESR), and C-reactive protein (CRP). Particular emphasis was placed on the white blood cell count (WBC), ESR, and CRP. A preoperative joint aspiration was also performed and sent to the lab for a cell count with differential, gram stain, culture with specificity, and crystal analysis. The final preoperative PJI diagnosis was made in accordance with the 2011 version of the Musculoskeletal Infection Society (MSIS) criteria. This definition includes one of the two major criteria being met or three of the five minor criteria being met. The two major criteria include: (1) sinus tract communicating with the prosthesis (2) pathogen isolated by culture from two separate tissue/fluid samples from the affected joint. The five minor criteria include: (1) elevated ESR (>30mm/h) and CRP (>10mg/L); (2) elevated synovial WBC (>3,000cells/uI) or positive change on leukocyte esterase test strip; (3) elevated synovial polymorphonuclear leukocytes (PMNs) >80%; (4) pathogen isolation in one culture; and (5) intraoperative frozen section with greater than five PMNs per high-power field (hpf) in 5 hpf at 400 magnification.

Surgical technique

Preparation/Positioning

Under general endotracheal anesthesia, the patient is positioned lateral decubitus with the operative side up and held in this position with the McGuire hip positioner. The hip is appropriately prepped and draped in the usual sterile fashion.

Approach

**Figure 2:**
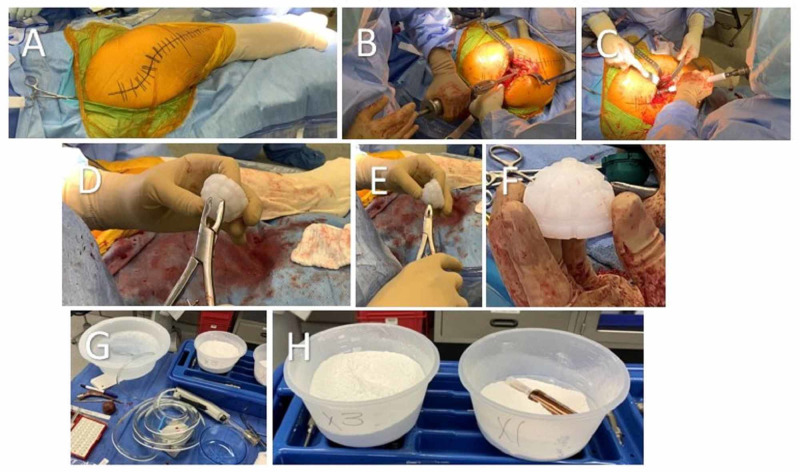
(A) Marked incision using primary total hip arthroplasty (THA) skin incision; (B) Femoral extractor is back slapped to remove primary femoral component; (C) The acetabulum is reamed progressively upwards until appropriate fit is found. In this example the acetabulum is reamed from 46 mm to 52 mm; (D, E, F) The back of the polyethylene acetabulum liner is shaped with a rounger to increase the surface area for cement integration. In this example a Zimmer Trilogy 56 mm x 36 mm liner is used; (G) 3 grams of Chlorpactin WCS-90 are mixed in 3 liters of normal saline in a basin on the back sterile table. This solution is used to irrigate the hip intermittently throughout the procedure; (H) The pre-mixed batches of cement with impregnated antibiotics. The cement on the left (labeled X3) contains 3 batches of cement with 6 grams of vancomycin powder and 7.2 grams of tobramycin powder and is used in the femoral canal. The cement on the right (labeled X1) contains 1 batch of cement with 2 grams of vancomycin powder and 2.4 grams of tobramycin powder and is used in the acetabulum.

All operations were performed with an anterolateral approach (Watson-Jones) by the same senior surgeon (MB), who is experienced in hip revision and infection treatment. A longitudinal lateral skin incision is performed using the old incision via an ellipse of skin (Figure 2a). The soft tissue is dissected down developing an interval between the tensor fasciae latae and gluteus medius. The anterior one-third of the hip abductors are taken off the trochanter to expose the anterior capsule. Once partial detachment of the hip abductors is achieved, a T-capsulotomy is performed. 

Intraoperative Cultures and Intraoperative Frozen Section

Cultures are obtained from the hip joint, the femur, and the acetabulum. An intraoperative frozen section is obtained and sent to the in-house pathologist for number of polymorphonuclear leukocytes per high-power field. Confirmation of infection is confirmed by intraoperative frozen section with greater than five PMNs per hpf in 5 hpfs at 400 magnification prior to new implant insertion.

Implant Removal

The hip is then dislocated anteriorly and the femoral head is removed. The mode of femoral stem removal is determined by the implant type and whether the stem is solidly fixed or loose. On occasion, if the femoral component is noted to be solidly fixed, an extended trochanteric osteotomy (ETO) may be performed. However, during this example a combination of the Midas Rex with a pencil tip burr and flexible osteotomes are used to dissect the bony integration. The extractor is then placed on the femoral component and removed by back slapping the femoral extractor (Figure 2b).

Then attention is turned to the acetabulum. The acetabulum is completely dissected out removing all soft tissue surrounding the acetabular rim. The acetabular component is removed using the short-blade on the explant instrument.

Debridement

This step involves removal of all necrotic and inflammatory bone, synovium, and soft tissue. A combination of a rongeur and curettes are used to remove all visibly infected tissue. Additional debridement of the femoral canal is achieved by using reverse hooks. The emphasis of this step is to obtain a surgical margin free of necrotic tissue to minimize the risk of infection recurrence.

Acetabulum and Femoral Preparation

The acetabulum is then reamed progressively upwards until an appropriate fit is found for a cement mantle thickness of 2 to 4 mm (Figure 2c). A trial is used to determine the appropriate fit in the acetabulum for the cemented polyethylene component. In this example the Zimmer Trilogy 56 mm x 36 mm liner is used. This polyethylene acetabulum liner is obtained and the back of this liner is shaped with a rounger to increase the surface area for cement integration (Figure 2d-2f).

Attention is now turned to the femur. The femoral canal is reamed progressively upward leaving cancellous bone for future cement integration. Once again a cement mantle thickness of 2 to 4 mm is recommended. A trial is used to confirm the appropriately sized construct, a 13 x 170 mm cemented revision calcar (CRC) stem with a 10 mm calcar augment to compensate for medial calcar bone loss is deemed appropriate in this example.

Irrigation and Acetabulum Prosthesis Implantation

Three liters of normal saline is used to completely irrigate the hip. Then 3 grams of Clorpactin WCS-90 are mixed in 3 liters of normal saline in a basin on the back sterile table, approximately one-third (1 liter) of this solution is irrigated into the hip (Figure 2g). The hip is suctioned of fluid and the acetabulum is dried and packed with a lap sponge.

**Figure 3:**
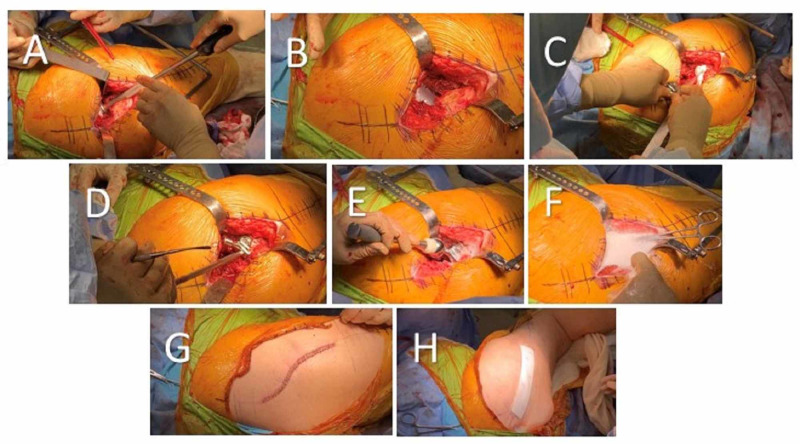
(A, B) The newly shaped polyethylene acetabulum liner is inserted into the cement mantle and held at approximately 40 to 45 degrees of abduction and 10 to 12 degrees of anteversion until the cement is completely hardened; (C, D) The femoral stem is inserted into the femoral canal, in this example a Zimmer VerSys 13 x 170 mm CRC stem with 10 mm calcar augment is used; (E) The femoral head is impacted onto the morse taper. In this example a Zimmer VerSys 36 +3.5 mm metal femoral head is used; (F) The hip is irrigated with the Clorpactin solution and left in place for a couple of minutes prior to closure; (G) Closure including skin approximation with staples; (H) Optifoam silver occlusive dressing over wound.

The first batch of cement is mixed using one batch of cement with 2 grams of vancomycin powder and 2.4 grams of tobramycin powder (Figure 2h). This cement is then packed into the acetabulum. The Zimmer Trilogy 56 mm x 36 mm liner is inserted into the cement mantle and impacted in place. All excess cement is removed. This is liner is held in place at approximately 40 to 45 degrees of abduction and 10 to 12 degrees of anteversion until the cement is completely hardened (Figure 3a-3b). 

Irrigation and Femoral Prosthesis Implantation

The femoral canal is irrigated with approximately 1 liter of the Clorpactin solution that was previously prepared on the back table. The femoral canal is suctioned of fluid until dry.

The second batch of cement is mixed using three batches of cement with 6 grams of vancomycin powder and 7.2 grams of tobramycin powder. The Zimmer VerSys 13 x 170 mm CRC stem with 10 mm calcar augment is assembled and tightened on the back table. The cement is then finger packed into the femoral canal. The stem is inserted in approximately 5 degrees of anteversion, impacted down onto the remaining medial calcar and any excess cement is removed. The stem is held in place until the cement is completely hardened (Figure 3c-3d). 

Trial reductions are performed until a trial head is found to have good stability and soft tissue tensioning. The hip is tested to ensure it’s stable in flexion and extension. In this example a 36 +3.5 mm trial head was deemed appropriate. The hip is dislocated, trial head removed, and femoral Morse taper is cleaned. A Zimmer VerSys 36 +3.5 mm metal femoral head is impacted onto the taper (Figure 3e).

The acetabulum is further irrigated and suctioned using a Clorpactin irrigation solution to remove any debris. The hip is then reduced and stability is confirmed.

Final Irrigation and Closure

The hip is further irrigated with the remaining Clorpactin solution of approximately 1 liter, some of this solution is left in place for a one minute soak (Figure 3f). Lastly the solution is suctioned out prior to closure.

**Figure 4:**
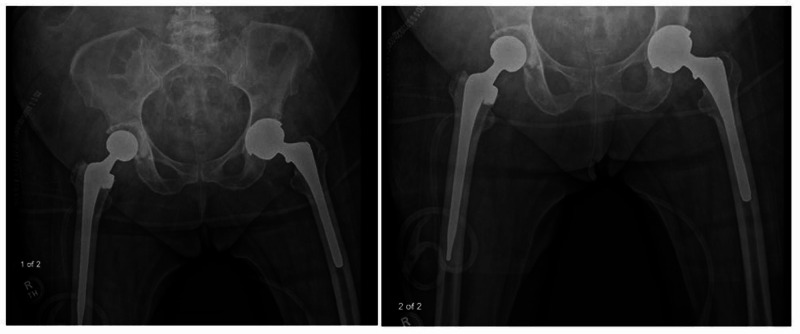
Final postoperative plain radiographs demonstrating this alternative one-stage exchange arthroplasty technique on the right hip.

After assuring adequate hemostasis, a 1/4 inch Hemovac drain is placed into the hip joint. The capsule is closed with interrupted #1 Ethibond and the abductors are reapproximated to the trochanter with interrupted #1 Ethibond. The fascia lata is closed with a running #1 Ethibond. The deep subcutaneous layer is closed with interrupted #1 Vicryl and the superficial subcutaneous tissue is closed with interrupted 2-0 Vicryl. The skin is approximated with staples (Figure 3g). The wound is dressed with Optifoam silver occlusive dressing and the drain site is dressed with a Biopatch and 4 x 4's (Figure 3h). Lastly, the patient is placed into an abduction pillow and plain radiographs are taken in the post-anesthesia care unit (PACU) (Figure 4).

Postoperative Protocol and Antibiotics

Our typical primary THA postoperative protocol is followed including weight-bearing as tolerated to the operative extremity with physical therapy starting postoperative day one. Initial antibiotics are chosen based on pre-operative culture results. This antibiotic regimen is then specifically tailored based on intra-operative culture results and infectious disease physician recommendations. Patients typically remain on outpatient intravenous antibiotics for six weeks based on culture sensitivities. However, the duration may change under infectious disease physician recommendations and trending lab values that include WBC, CRP, ESR, and nutrition markers.

Statistical analysis

The odds of re-infection, need for additional surgery, and retention of a stable infection-free implant were evaluated amongst the patients. The statistical significance was assessed at a confidence interval of 95% and the analysis was performed by using a two-sided confidence interval for a single proportion [15].

## Results

Of the eight patients, three were female and five were male with a mean age of 70.5 years (SD 11.2, range 53-87). Three of the patients were diabetics and three were current or prior smokers. The mean BMI was 26.12 (range 16.72-43.46) and the mean interval between prior surgery and the one-stage exchange arthroplasty was 46 months (range 2-144).

**Figure 5:**
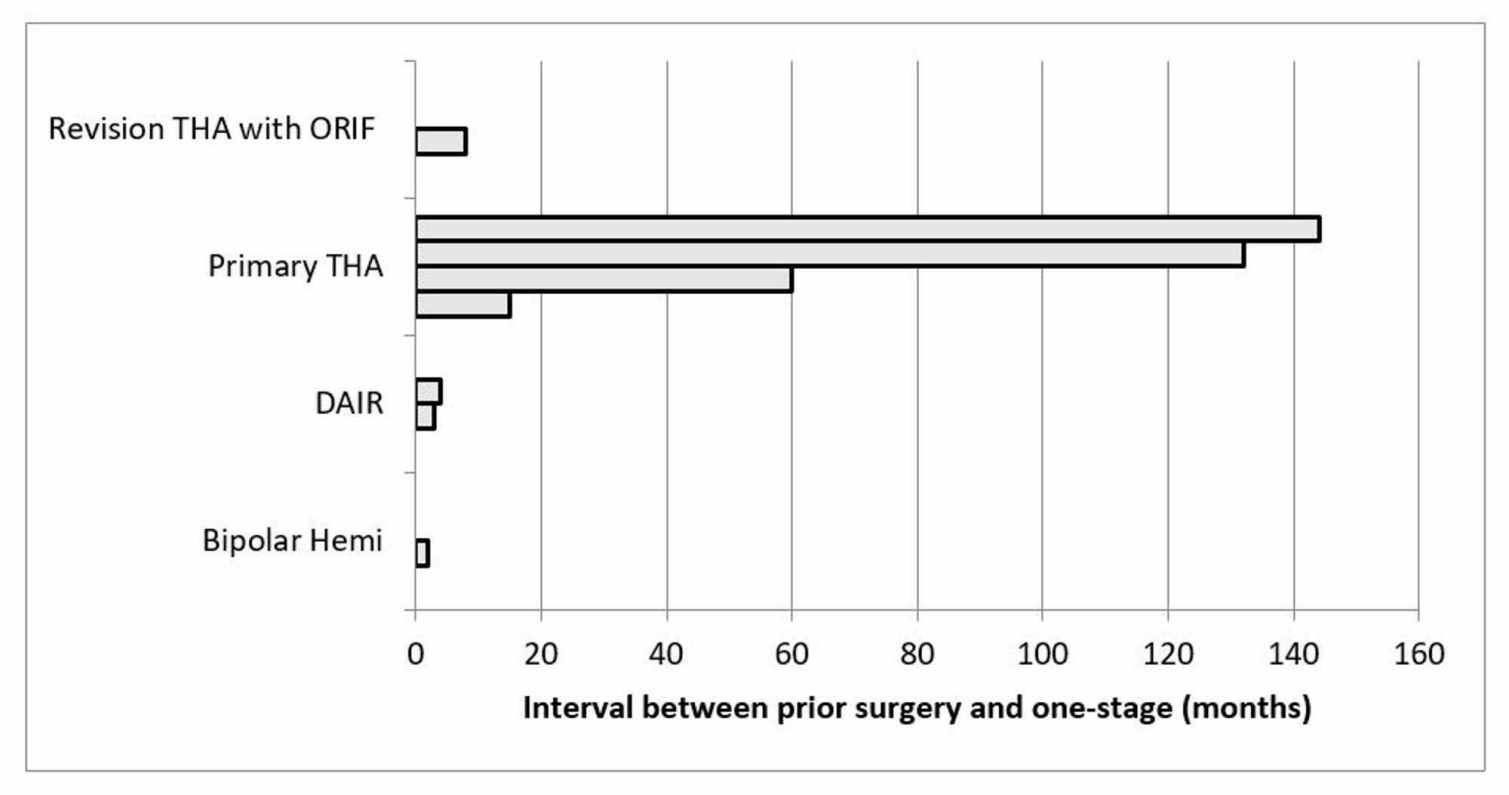
Patient summary of the interval between prior surgery and one-stage exchange total hip arthroplasty (THA), organized by prior surgery type. ORIF = open reduction internal fixation; DAIR = debridement, antibiotics, and implant retention

Patients who had a prior DAIR (Debridement, Antibiotics, and Implant Retention) surgery performed were more likely to require a one-stage procedure sooner than if the patients had a prior primary THA performed (Figure 5). Six patients (75%) had infection eradication with retention of a stable implant and no additional surgery at a mean follow-up of 35.7 months (range 17-50). One patient (12.5%) underwent closed reduction for a dislocated THA at one month; however this patient remained infection-free at the most recent follow-up of 41 months. One patient (12.5%) who was the oldest patient (87 years) died 18 days postoperatively. Of note, this deceased patient did have a past medical history significant for prior myocardial infection, prior cerebral vascular accident, chronic obstructive pulmonary disease, hypertension, hyperlipidemia, gastroesophageal reflux disease, and dementia.

Overall, all living patients (87.5%) retained their one-stage exchange THA. One patient (12.5%, CI 95% 0.3-52.7) required additional surgery in the form of a closed reduction and zero patients (0.0%, CI 95% 0.0-36.9) required additional open surgery. 

## Discussion

Although THA infection rates are at an all-time low with reports between 1 and 3% [2,3], the exponential increase in primary arthroplasty volume possesses a significant increase in healthcare costs with greater patient morbidity. As such, there has been a growing interest in the optimal treatment strategy for prosthetic joint infections. Whether a one-stage or two-stage exchange is performed, the goal of infection eradication with retention of a stable implant remains the same. Our study described an alternative one-stage technique and reported initial results from a small case series of patients. We found that all living patients (87.5%) retained their one-stage exchange THA.

A two-stage exchange is currently considered the “gold standard” [10]. However, successful eradication of infection is achievable with a one-stage exchange. A recent retrospective study reported a 76.9% overall survival rate with 96.2% infection control at a minimum of 10-year follow-up for a one-stage exchange THA [16]. Svensson et al. analyzed the risk of re-revision in infected primary THAs treated with a one-stage (n=404) or two-stage (n=1250) exchange. They found no significant difference in overall survival rate, revision due to all causes, infection, or aseptic loosening [17]. A large systematic review found a rate of reinfection to be 8.6% for one-stage and 10.2% for two-stage infected THA revisions [18].

Patient selection must be considered when determining the treatment strategy for prosthetic joint infections. Thakrar et al. concluded that a one-stage exchange is an acceptable form of surgical treatment in patients without severe immunocompromise, significant soft-tissue or bony compromise, or concurrent acute sepsis [13]. Another study reported that the absence of wound complications after the initial total hip replacement, good general health of the patient, infecting organism of methicillin-sensitive *Staphylococcus epidermidis*, *Staphylococcus aureus*, or *Streptococcus* species, and an organism sensitive to the antibiotic mixed into the bone cement were factors associated with a successful one-stage exchange THA [19]. This study also reported that polymicrobial infection, gram-negative organisms, especially *Pseudomonas* species, and certain gram-positive organisms such as methicillin-resistant *Staphylococcus epidermidis* and Group D *Streptococcus* were factors associated with failure.

There are many advantages of a one-stage exchange arthroplasty. Simply performing the explanation and reimplantation in one procedure offers less morbidity, mortality, and functional impairment to the patient [14]. This improved functional outcome has been reported and continues to be investigated in Harris hip scores, Oxford hip scores, UCLA activities scores, and Western Ontario and McMaster Universities Arthritis Index (WOMAC) scores [16,20-22]. A one-stage procedure also decreases cost for the patient and the health care system. Kouche et al. reported that THA revisions performed for infection cost 3.6 times more than a primary THA [23]. They concluded that THA infections cause a large economic impact with extra costs due to extended hospital stays and longer rehabilitation time. Thus, a one-stage exchange improves overall cost through a single operative procedure, fewer antibiotics, and reduced hospitalization time [24].

There are a variety of limitations to our study. First, the study took place at a single institution with a small sample size of patients. This makes it difficult to develop a conclusion of great statistical significance. Although, using a single institution with the same primary surgeon minimizes intraoperative surgical technique variability. Second, this study was retrospective in nature without a two-stage exchange control group. Also, there were confounding factors such as obesity, smoking status, and diabetes mellitus that may have affected our results. Regardless of our limitations, we do believe the results provide a reference for surgeons to consider when treating the chronically infected total hip. This study will hopefully encourage future randomized prospective studies with a larger sample size.

## Conclusions

Based on the results of our case series and citied published reports, single-stage exchange arthroplasty may be a useful option for the treatment of chronic hip PJIs. Our case series provides evidence that infection eradication and function preservation are possible using our one-stage exchange arthroplasty technique that does not include the use of a metal cup. Therefore, an antibiotic-impregnated cemented femoral stem with antibiotic-impregnated cemented polyethylene acetabular liner could be considered as an alternative treatment method in the chronic infected total hip. However, a multi-center study with randomization is necessary to further validate our results. 
